# Dual-Responsive Reconfigurable Miniature Fiberbots: A Study for Vascular Embolization

**DOI:** 10.34133/research.0404

**Published:** 2024-07-15

**Authors:** Xurui Liu, Xin Song, Li Zhang

**Affiliations:** ^1^ Department of Mechanical and Automation Engineering, The Chinese University of Hong Kong, Shatin, New Territories, Hong Kong SAR, China.; ^2^ Department of Surgery, The Chinese University of Hong Kong, Shatin, Hong Kong SAR, China.

## Abstract

Navigating the intricate and narrow vascular pathways of the body remains a formidable challenge in vascular embolization, often limiting the maneuverability and steerability of traditional catheters. This study, by T.T. Xu and co-workers, introduces dual-responsive reconfigurable miniature fiberbots, which are capable of catheter-assisted deployment, navigation, and embolization in vascular systems. Through meticulous design and magnetic control, this work successfully validates a multistage vascular embolization approach in the renal artery of rabbits in vivo. The experiments not only overcome the existing limitations of conventional catheterization techniques but also open new avenues for minimally invasive treatments.

Catheter-based interventions have substantially advanced minimally invasive diagnostics and treatments, offering extensive clinical benefits [1]. Yet, reaching the distal vascular system presents notable challenges. Factors such as elongated access paths, vessel tortuosity, and the fragility of blood vessel walls complicate catheter navigation [2]. Interventional procedures typically involve inserting catheters through the femoral artery into the convoluted distal small vessels. This intricate surgery demands constant surgeon vigilance and skill, posing risks like vessel wall damage and increased procedural durations, thus heightening procedural risks. These challenges are particularly acute when accessing distal cortical arteries, which are essential for managing conditions like aneurysms, arteriovenous malformations, and various tumors [3]. In recent years, there has been a growing focus on developing miniature machines capable of reaching challenging body sites due to their untethered design and small size [4–6]. These devices are valued for their ability to navigate narrow and confined spaces. Magnetic actuation is especially notable for wireless control of microrobots due to safe and fast interactions [7]. However, previous designs have struggled to meet clinical needs, lacking essential features such as multi-function integration, real-time tracking, and the creation of robotic or intelligent platforms for use in the operating room.

To address these challenges, a novel approach was introduced involving the use of thermal and magnetic dual-responsive shape memory microrobots (SMMs) for complex vascular embolization tasks [8], as depicted in Figure. This method significantly improves the efficiency of in vivo embolization processes by providing faster delivery and better access to blood vessels. Composed of an organo-gel matrix embedded with ferromagnetic particles, these SMMs are biocompatible and clearly visible under fluoroscopy imaging. Moreover, the SMMs quickly respond to thermal and magnetic stimuli while maintaining excellent shape memory properties. At an initial temperature of 20 °C, the SMMs are straight in shape and can be easily inserted into a catheter. Upon reaching the warmer blood temperature of 38 °C, the robot rapidly (within 2 s) transforms into a pre-programmed helical shape, enabling effective navigation through narrow and winding blood vessels via rotating magnetic fields. The effectiveness of this multistage embolization strategy was confirmed in the renal artery of rabbits in vivo. Post-operation results indicated a 36% reduction in the volume of the embolized kidney.

**Figure.**
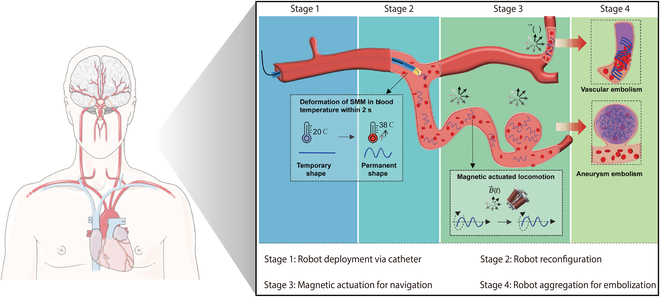
Dual-responsive reconfigurable miniature fiberbots for multistage vascular embolization.

Taken together, this innovation offers the clinical potential for more efficient therapy delivery, advanced medical imaging techniques, and improved treatment outcomes. Robotic embolization represents a groundbreaking vision, aiming to execute endovascular procedures using wirelessly controlled robots [9–12]. However, transforming this vision from bench to bedside and bridging the gap between experimental success and practical utility necessitate substantial advancements. Future translational research must prioritize the development of materials that not only ensure biocompatibility for long-term embolization but also enhance the long-term stability of the embolization process. Enhancing the robots, design to include protective coatings, such as those demonstrated with hydrogel layers, represents a critical step toward minimizing toxicity and ensuring patient safety. Furthermore, the hydrogel layer can be engineered with specific physical and chemical properties to enhance embolization efficiency. For instance, robots coated with a swellable hydrogel expand in volume when deployed at the targeted embolization site, improving the filling rate. Another strategy involves coating the robots with thrombin or other drugs to promote localized thrombosis, thereby further enhancing embolization efficiency. Additionally, a bioadhesive mechanism can be incorporated into the hydrogel layer to create a strong bond between the robots and the blood vessel wall, preventing their passive drift due to dynamic blood flow.

Achieving precise control and navigation of these robots within the intricate and narrow confines of human vasculature also requires the integration of advanced imaging modalities. These modalities provide the high-resolution guidance necessary for maneuvering the robots through submillimeter blood vessels. Ultimately, the successful translation of robots into clinical practice will depend on addressing these challenges, underscoring the need for a multidisciplinary approach that combines materials science, biomedical engineering, robotics, and medical imaging to realize the full potential of robotic embolization technologies.
